# Is There a Place for Apheresis in the Management of Idiopathic Membranous Nephropathy? A Report of Three Cases and Literature Review

**DOI:** 10.3390/jpm14030249

**Published:** 2024-02-26

**Authors:** Hamza Naciri Bennani, Augustin Twite Banza, Diane Giovannini, Lionel Motte, Johan Noble, Alexandra Corbu, Paolo Malvezzi, Thomas Jouve, Lionel Rostaing

**Affiliations:** 1 Nephrology, Haemodialysis, Apheresis and Kidney Transplantation Department, Grenoble University Hospital, 38043 Grenoble, France; hnaciribennani@chu-grenoble.fr (H.N.B.); augustinbanza63@gmail.com (A.T.B.); lmotte@chu-grenoble.fr (L.M.); jnoble@chu-grenoble.fr (J.N.); acorbu@chu-grenoble.fr (A.C.); pmalvezzi@chu-grenoble.fr (P.M.); tjouve@chu-grenoble.fr (T.J.); 2 Pathology Laboratory, Grenoble University Hospital, 38043 Grenoble, France; dgiovannini@chu-grenoble.fr; 3 Faculty of Medicine, Grenoble Alpes University, 38043 Grenoble, France

**Keywords:** idiopathic membranous nephropathy, apheresis, double-filtration plasmapheresis, semi-specific immunoadsorption, rituximab

## Abstract

Membranous nephropathy constitutes approximately 20% of adult nephrotic syndrome cases. In approximately 80% of cases, membranous nephropathy is primary, mediated by IgG autoantibodies primarily targeting podocyte antigens (PLA2R, THSD7A, etc.). The treatment involves a combination of corticosteroids and cyclophosphamide or anti-CD20-based therapies, e.g., rituximab. In the event of significant proteinuria and in order to avoid the urinary elimination of rituximab, therapeutic apheresis, in particular semi-specific immunoadsorption, may be an option allowing for a reduction in proteinuria and autoantibodies before initiating treatment with rituximab. We present the preliminary experience of three patients treated with semi-specific immunoadsorption for primary membranous nephropathy between January 2021 and March 2023. Two patients were anti-PLA2R-autoantibody-positive and one was seronegative. The average age was 59 ± 17 years. Semi-specific immunoadsorption did not reduce albuminuria, but it, nevertheless, led to an increase in serum albumin, contributing to the regression of edema. It effectively eliminated anti-PLA2R autoantibodies in the two anti-PLA2R-positive patients. Consequently, apheresis may not induce a rapid reduction in proteinuria, but could contribute to a more accelerated remission when combined with the anti-CD20 treatment.

## 1. Introduction

Membranous nephropathy is a glomerular disorder characterized histologically by the thickening of the glomerular basement membrane due to granular deposits of IgG and complement fractions [1]. It constitutes approximately 20% of adult nephrotic syndrome cases, typically diagnosed between the ages of 30 and 50 [2,3].

In approximately 80% of cases, membranous nephropathy is primary [4], mediated by IgG autoantibodies primarily targeting podocyte antigens. The most prevalent among these is the antibody directed against the M-type phospholipase A2 receptor (PLA2R), identified in 75 to 85% of patients with primary membranous nephropathy [5,6]. Recent discoveries include other target molecules, such as thrombospondin-type-1-domain-containing 7A (THSD7A), neural epidermal growth factor type 1 protein (NELL-1), semaphorin 3B (SEMA3B), superoxide dismutase 2 (SOD2), and aldose reductase (AR) [7].

The conventional treatment involves a combination of corticosteroids and cyclophosphamide, following the Ponticelli protocol, despite its significant side effects (infection, osteoporosis, hemorrhagic cystitis, infertility, cancers, etc.) [8,9]. The recognition of the autoimmune nature of membranous nephropathy has prompted the adoption of anti-CD20-based therapies, e.g., rituximab, which are characterized by their lower toxicity and greater specificity when compared to alkylating agents and calcineurin inhibitors [7].

The use of rituximab, an antibody targeting CD20 on B cells, facilitates remission in 60 to 80% of cases of idiopathic membranous nephropathy [10]. In 23 to 43% of cases, the presence of anti-rituximab antibodies has been identified in patients undergoing treatment for membranous nephropathy, posing a substantial risk of relapse [10]. In addition, resistance to rituximab may be attributed, in part, to the drug’s diminished bioavailability, a consequence of significant albuminuria in membranous nephropathy leading to the loss of rituximab in the urine [11]. Indeed, in patients with nephrotic syndrome and non-selective proteinuria, the rituximab pharmacokinetics are altered profoundly, and rituximab does not maintain high enough levels for a sufficiently long period of time, which may render the rituximab treatment ineffective. In such instances, therapeutic apheresis, specifically, semi-specific immunoadsorption, emerges as a viable option to mitigate proteinuria by reducing autoantibodies before initiating rituximab therapy; indeed, semi-specific immunoadsorption is capable of eliminating approximately 60% of circulating IgG after a single session, treating 100 mL/kg of plasma volume [12]. Here, we present the preliminary experience of three patients treated with semi-specific immunoadsorption for primary membranous nephropathy.

## 2. Materials and Methods

Data were retrospectively gathered from three patients diagnosed with primary membranous nephropathy who underwent the semi-specific immunoadsorption treatment between January 2021 and March 2023. The diagnosis of primary membranous nephropathy was established through a renal biopsy and the exclusion of all secondary causes. All patients received an optimal symptomatic treatment, including renin–angiotensin system antagonists, lipid-lowering agents, and anticoagulants, if deemed necessary.

The primary endpoint aimed at evaluating the reduction in albuminuria from the initiation to the conclusion of the semi-specific immunoadsorption sessions. Secondary endpoints included the clinical response, hemodynamic tolerance during the course of the immunoadsorption sessions, and changes in serum albumin levels. Various biological parameters were recorded for each patient, encompassing serum creatinine, IgG, IgM, IgA, serum albumin levels, and albuminuria. Anti-PLA2R antibody assessments were conducted for all three patients, with anti-THSD7A antibody testing performed for the patient negative for anti-PLA2R autoantibodies.

During the semi-specific immunoadsorption, patient plasma was separated from blood cells via centrifugation (utilizing Spectra Optia^®^-Terumo BCT, Lakewood, CO, USA or Com.Tec^®^ generators, Fresenius Kabi, Bad-Homburg, Germany). Subsequently, the plasma underwent a treatment using two Globaffin^®^ columns operating consecutively (Fresenius Medical Care, Bad-Homburg, Germany); thereafter, after being treated, i.e., all the IgG-depleted plasma was reinfused into the patient. In addition, no albumin was infused into the patient. These columns employed the synthetic peptide GAM, exhibiting a strong affinity for the constant fraction of IgG subclasses 1, 2, and 4, and a weaker affinity for IgG subclass 3, IgA, and IgM [13]. The plasma volume treated in one immunoadsorption session was 100 mL/kg. Vascular access necessitated the insertion of a central venous jugular catheter, which was removed upon the completion of the apheresis therapy. Extracorporeal anticoagulation was achieved through regional anticoagulation using citrate, with calcium reinjection into the returning venous line [14].

### 2.1. Statistical Analyses

Clinical and biological data were extracted from electronic medical and monitoring records gathered during the immunoadsorption sessions. Statistical analyses were conducted using Excel 2016 software, and the results were presented as mean ± SD or medians (ranges), as appropriate.

### 2.2. Ethics

Ethical approval was not required for this study, in accordance with local or national guidelines, i.e., these were 3 case reports. Written informed consent was obtained from the patients for the publication of the details of their medical cases.

## 3. Results

**Figure 1:**
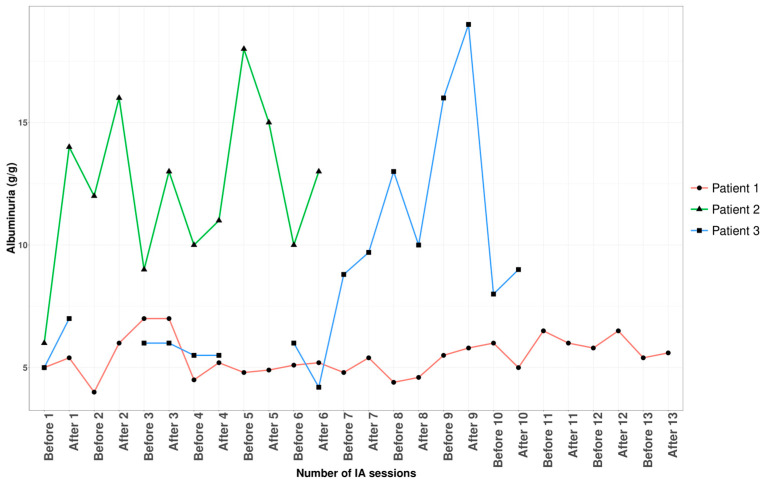
Outcomes of albuminuria before and after apheresis sessions.

**Figure 2:**
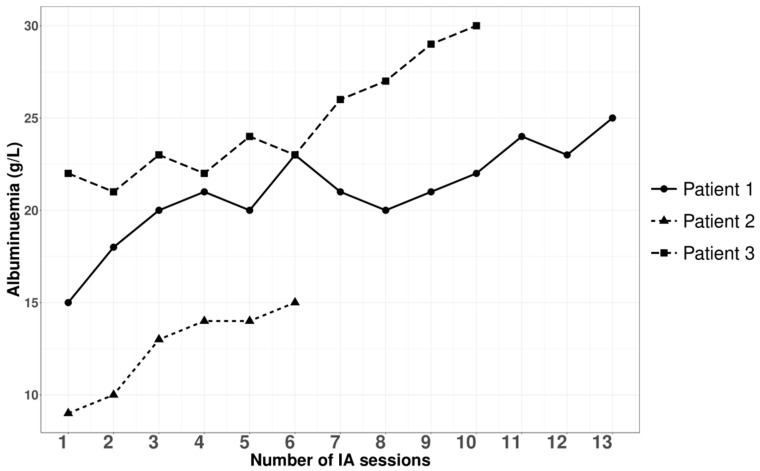
Outcomes of albuminemia during and after apheresis sessions.

**Figure 3:**
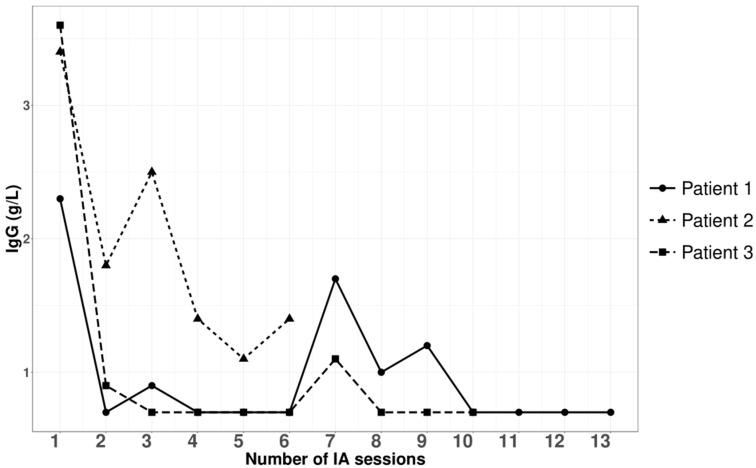
Outcomes of serum immunoglobulin G during and after apheresis sessions.

**Figure 4:**
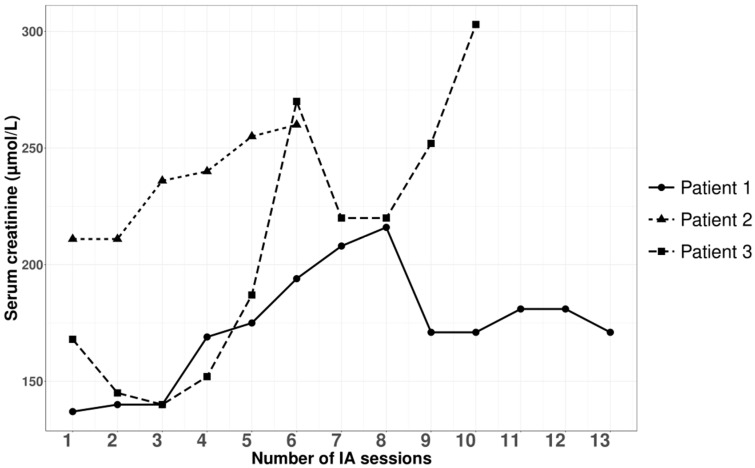
Outcomes of serum creatinine levels during and after apheresis sessions.

Three patients with nephrotic syndrome related to primary membranous nephropathy were treated with semi-specific immunoadsorption, of which two were anti-PLA2R-autoantibody-positive and one seronegative. The average age was 59 ± 17 years, with a male-to-female sex ratio of 2:1. Hemodynamic tolerance during the immunoadsorption sessions was good. No infectious complications were documented during or within a month after the apheresis procedures. Figure 1, Figure 2, Figure 3 and Figure 4, and Supplementary Figures S1 and S2 illustrate the progression in our patients during the immunoadsorption sessions, representing albuminuria, serum albumin, IgA, IgM, IgG, and serum creatinine, respectively.

### 3.1. Patient 1

A 41-year-old woman with a history of hypertension was admitted to the intensive cardiac care unit in October 2022 due to malignant hypertension. A left renal vein thrombosis was discovered, revealing nephrotic syndrome with a serum albumin concentration of 15.8 g/L and albuminuria at 8 g/24 h, complicated by acute renal failure, with creatinine levels escalating from 44 µmol/L to 142 µmol/L. No secondary cause was identified. An immunological assessment revealed positive anti-PLA2R autoantibodies at 334 IU/mL. A kidney biopsy unveiled stage two membranous nephropathy associated with focal and segmental glomerulosclerosis (FSGS) lesions and IF/TA (interstitial fibrosis/tubular atrophy) lesions (40%). Immunofluorescence staining was positive for anti-PLA2R, -IgG1(+), -IgG2(+++), -IgG3(++), and -IgG4(+++). In this instance, the decision was to initiate treatment with semi-specific immunoadsorption before introducing rituximab therapy. The patient underwent a total of 13 immunoadsorption sessions from 1 December 2022 to 21 December 2022. Rituximab 1 g was administered on 12 December 2022 and 27 December 2022, respecting a 3-day interval between the first rituximab infusion and the subsequent immunoadsorption session. The level of anti-PLA2R autoantibodies decreased from 334 IU/mL to 117 IU/mL between the initiation and conclusion of the immunoadsorption sessions. However, immunoadsorption did not result in a reduction in albuminuria. Renal function experienced a slight deterioration, with serum creatinine progressing from 137 µmol/L to 171 µmol/L. Conversely, there was an increase in albuminemia from 15 g/L to 25 g/L. After a 12-month follow-up, the patient was in partial remission, with a serum albumin concentration of 27 g/L, an ACR (albumin-to-creatinine ratio) of 2 g/g, and a serum creatinine level of 122 µmol/L, i.e., an estimated glomerular filtration rate (eGFR) of 48 mL/min/1.73 m^2^.

### 3.2. Patient 2

A 76-year-old man with a history of hypertension was admitted to nephrology on 25 November 2022 due to nephrotic syndrome, presenting with a serum albumin concentration of 15 g/L and an ACR of 10 g/g, complicated by acute renal failure with a serum creatinine level of 132 µmol/L. A kidney biopsy revealed stage two membranous nephropathy with negativity for anti-PLA2R and positivity for anti-IgG1(++), -IgG2(+++), -IgG3(+++), and -IgG4(+++) on immunofluorescence staining. Anti-PLA2R and anti-THSD7A autoantibodies were both negative, and no secondary cause was identified, i.e., normal PET-CT, negative HBV, HCV, syphilis, and HIV serologies, normal serum complement levels, and no detectable autoantibodies. Symptomatic treatment was initiated, resulting in an improvement in serum creatinine to 105 µmol/L and a weight loss of 7 kg.

One week after discharge from the hospital on 4 January 2023, despite optimal symptomatic treatment, the patient’s nephrotic syndrome worsened (serum albumin concentration of 8 g/L and ACR of 10 g/g), and his creatinine level increased to 200 µmol/L. Faced with this situation, semi-specific immunoadsorption therapy was initiated. The patient underwent six immunoadsorption sessions from 6 January 2023 to 12 January 2023. Due to the lack of a response to immunoadsorption and the persistence of significant albuminuria at 10 g/g after the sixth session, tacrolimus therapy (target trough levels between 3 and 5 ng/mL) was introduced along with oral corticosteroids (1 mg/kg/day).

After an 8-month follow-up, the patient was in partial remission, with a serum albumin concentration of 41 g/L, an ACR of 2.1 g/g, and a serum creatinine level of 180 µmol/L (eGFR at 30 mL/min/1.73 m^2^). The tacrolimus trough level was 7.8 ng/mL.

### 3.3. Patient 3

A 61-year-old man with a history of type 2 diabetes was treated for nephrotic syndrome secondary to a PLA2R-positive primary membranous nephropathy since December 2019. Initially, the anti-PLA2R autoantibody titer was at 1500 IU/mL. He underwent five courses of rituximab (1 g per infusion) from 8 January 2020 to 28 August 2020, achieving partial remission with a serum creatinine level of 131 µmol/L (eGFR = 51 mL/min/1.73 m^2^), ACR of 6 g/g, and negative anti-PLA2R autoantibodies. A kidney biopsy, performed in December 2019, revealed stage two membranous nephropathy with negativity for anti-PLA2R and positivity for anti-IgG1(++), -IgG2(+/−), -IgG3(++), and -IgG4(+++) on immunofluorescence staining.

Following a relapse of nephrotic syndrome in May 2021 (ACR 5 g/g, serum albumin concentration of 22 g/L, serum creatinine level of 168 µmol/L, anti-PLA2R at 249 IU/mL, and anti-THSD7A negative), anti-rituximab antibodies were monitored and found to be negative. Semi-specific immunoadsorption sessions were initiated, and the patient underwent 10 sessions from 3 May 2021 to 13 May 2021. Although the immunoadsorption sessions resulted in an improvement in the serum albumin concentration to 30 g/L and a decrease in the anti-PLA2R level to 16 IU/mL, significant albuminuria persisted (ACR of 8 g/g) and renal function declined (creatinine 303 µmol/L). In the absence of a response to immunoadsorption treatment, obinutuzumab therapy was introduced.

After a 3-year follow-up, the patient exhibited partial remission with albuminemia at 27 g/L, an ACR of 2.8 g/g, and a serum creatinine level of 126 µmol/L (eGFR at 53 mL/min/1.73 m^2^).

## 4. Discussion

Despite the availability of treatment algorithms for idiopathic membranous nephropathy, determining an optimal individualized therapeutic approach remains challenging due to variations in disease progression severity and therapeutic response. While the majority of patients respond favorably to established immunosuppressive protocols, achieving remission is not guaranteed.

Rituximab has emerged as a primary therapeutic option for idiopathic membranous nephropathy, particularly following the identification of various pathogenic autoantibodies, predominantly anti-PLA2R and anti-THSD7A [15,16]. As per the 2021 recommendations from KDIGO, rituximab (RTX) is advised as the initial treatment for moderate-risk patients with a normal eGFR and significant proteinuria (exceeding 3.5 g/day). For high-risk patients characterized by a decreased eGFR, cyclophosphamide is recommended as the first-line treatment [7,17].

Rituximab, an anti-CD20 monoclonal antibody, induces B lymphocyte depletion, leading to a reduction in the production of autoantibodies implicated in membranous nephropathy [18].

Significantly, in instances of substantial albuminuria, a considerable portion of injected rituximab is eliminated in the urine [11]. Due to our patients presenting with heavy albuminuria, we employed semi-specific immunoadsorption to rapidly reduce culprit autoantibodies, aiming to promptly decrease albuminuria before introducing rituximab. The rationale behind this strategy lies in the association between the swift depletion of autoantibodies and a faster remission of membranous nephropathy [19,20,21,22,23] potentially impeding the progression of chronic kidney disease. Notably, Hoxha et al. [22] reported that patients with high anti-PLA2R autoantibody levels achieved remission later than those with lower autoantibody levels.

While our approach did not prove effective in reducing albuminuria in our three patients (Figure 1), it did result in an improvement in serum albumin, contributing to the regression of edema (Figure 2). The semi-specific immunoadsorption effectively eliminated anti-PLA2R autoantibodies in our two anti-PLA2R-positive patients.

Several published clinical cases have demonstrated the effectiveness of adding apheresis (double-filtration plasmapheresis (DFPP), plasma exchange) for patients with membranous nephropathy refractory to standard immunosuppressants, with a favorable evolution of nephrotic syndrome [23,24,25,26,27,28,29,30,31]. Various hypotheses, apart from the elimination of autoantibodies, were considered, including:

1. Elimination of LDL cholesterol: Enhancing the bioavailability of immunosuppressants (cyclophosphamide, calcineurin inhibitors, and steroids) and mitigating the glomerular toxicity associated with dyslipidemia [25,26].
2. Elimination of complement proteins and inflammatory cytokines: Supported by the efficacy of apheresis in seronegative membranous nephropathy [32]. Yabuuchi et al. [27] reported a case of a 61-year-old patient with idiopathic membranous nephropathy, primarily IgG4 on kidney biopsy, and treated unsuccessfully with corticosteroids, cyclophosphamide, and cyclosporine for 2 years. Following the initiation of DFPP at a rate of two sessions per month, partial remission was achieved, with proteinuria decreasing to less than 3 g/24 h after 4 years and less than 1 g/24 h after 9 years. No relapse occurred within 5 years post-DFPP.

Wen et al. [33] presented a case involving a 34-year-old man with nephrotic syndrome secondary to idiopathic anti-PLA2R-positive membranous nephropathy refractory to corticosteroids and calcineurin inhibitors. The patient underwent four plasma exchange sessions every other day and received two rituximab infusions on day 8 and day 28. This combined treatment led to complete remission of nephrotic syndrome (with the protein-to-creatinine ratio dropping from 8 to 0.3 g/g) and the achievement of negative anti-PLA2R autoantibodies after 8 weeks.

Hamilton et al. [34,35] conducted a prospective single-arm multicenter study, enrolling 12 patients with nephrotic syndrome due to primary membranous nephropathy and anti-PLA2R antibody levels >170 U/mL resistant after 6 months of symptomatic treatment. Two patients had previously received cyclophosphamide treatment 6 months before the initiation of the semi-specific immunoadsorption (IA). These patients underwent five daily IA sessions. The level of anti-PLA2R autoantibodies decreased from 702.5 [206.25–1089.75] IU/mL to 91 [31–120.5] IU/mL at the end of treatment, representing an average drop of 87% in antibody titers along with an average drop of 93% in IgG. Anti-PLA2R autoantibodies and IgG rebounded upon stopping IA before gradually decreasing during follow-ups, suggesting the potential need for combined therapy targeting B cells to avoid a rebound. These sessions resulted in an increase in serum albumin concentrations from 20.5 [19–21.25] g/L to 21.5 [19.25–25.25] g/L, 23 [20–24] g/L, and 25 [23–28] g/L after 12, 24, and 52 weeks, respectively (*p* < 0.001). However, there were no significant changes in proteinuria and renal function. Two patients from this cohort received a subsequent immunosuppressive treatment (cyclophosphamide, corticosteroids, and/or rituximab) due to the failure of the semi-specific immunoadsorption and worsening of nephrotic syndrome.

The outcomes of our three patients aligned with previous studies, indicating a delay between antibody reduction and the clinical response [15,23]. The persistence of nephrotic syndrome in our patients was expected, given the prolonged presence of anti-PLA2R autoantibodies in the glomerular basement membrane despite serum elimination through semi-specific immunoadsorption [36]. This phenomenon was attributed to the high affinity of anti-PLA2R autoantibodies for the podocyte, contributing to the extended duration of their presence in the glomerular basement membrane.

The anti-CD20 treatment, such as rituximab, may necessitate an extended duration exceeding one year to achieve clinical and biological remission. This timeframe is influenced by the half-life of the circulating autoantibodies and the repair time of the glomerular basement membrane [21]. In a rat model, the transplantation of kidneys affected by membranous nephropathy demonstrated a gradual reduction in proteinuria, which, although never reaching negative levels, showed a decreasing trend after 2 weeks post-transplantation. IgG deposits at the glomerular basement membrane only diminished significantly from the 28th week post-transplantation, disappearing after 40 weeks [37]. Consequently, apheresis may not induce a rapid reduction in proteinuria, but could contribute to a more accelerated remission when combined with the anti-CD20 treatment.

Supporting this notion, a study involving 10 patients with refractory nephrotic syndrome secondary to primary membranous nephropathy found that plasma exchange, along with intravenous immunoglobulin (IVIG) and rituximab, led to partial remission in 90% of patients with an average delay of 2.1 ± 0.5 months. This remission time was faster compared to patients treated with combinations of corticosteroids and cyclophosphamide or cyclosporine and corticosteroids, which took 7.4 ± 1.4 months and 6.4 ± 1.2 months, respectively (*p* < 0.05) [24]. Furthermore, the decrease in serum creatinine was significantly higher in patients treated with plasma exchange and rituximab compared to rituximab alone (*p* < 0.05).

In the GEMRITUX randomized controlled trial [38], including 77 patients with normal renal function receiving either rituximab or a continuation of the antiproteinuric treatment, a 65% remission rate in terms of proteinuria was observed in the rituximab group compared to 34% in the control group after 6 months of follow-up. Anti-PLA2R autoantibody titers were significantly reduced after three months in the rituximab group, emphasizing the importance of titer monitoring to predict the need for repeat infusions.

A retrospective cohort study comparing rituximab to glucocorticoids and oral cyclophosphamide, following a standardized protocol, showed no difference at 5 years in the rate of complete clinical remission, but a better safety profile in the rituximab-treated group [39]. The MENTOR trial [40], which included 130 patients treated with rituximab or cyclosporine, demonstrated that, at 12 months, 60% of the rituximab group and 52% of the cyclosporine group achieved complete or partial remission. Notably, at 24 months, 60% of the rituximab group remained in remission, while the remission rate in the cyclosporine group dropped to 20%. Among patients with anti-PLA2R-associated membranous nephropathy, the rituximab group exhibited a more rapid and greater decline in anti-PLA2R autoantibody titers.

Combining apheresis (semi-specific immunoadsorption) with the anti-CD20 treatment appears promising for accelerating the remission of nephrotic syndrome induced by membranous nephropathy. While the cost of semi-specific immunoadsorption could potentially be offset by reduced hospitalization and rituximab infusions, a prospective randomized study, especially in severe and refractory cases of membranous nephropathy, is warranted to evaluate the efficacy and cost-effectiveness of this combined approach.

## 5. Conclusions

In conclusion, the combination of rituximab and therapeutic apheresis as a first-line treatment for primary membranous nephropathy appears to be a promising therapeutic option. This approach has the potential to enhance the percentage of patients achieving remission and reduce the time required to attain this remission. Further research, particularly through prospective randomized studies, is essential to validate the effectiveness and assess the long-term outcomes and cost-effectiveness of this combined therapeutic strategy in the management of primary membranous nephropathy.

## Data Availability

All the data are available upon request from the corresponding author.

## Supplementary Materials

The following supporting information can be downloaded at: https://www.mdpi.com/article/10.3390/jpm14030249/s1, Figure S1: Outcome of serum immunoglobulin A during and after apheresis sessions; Figure S2: Outcome of serum immunoglobulin M during and after apheresis sessions.
