# Supplementary material for: Is There a Place for Apheresis in the Management of Idiopathic Membranous Nephropathy? A Report of Three Cases and Literature Review
Source: J Pers Med. 2024 Feb 26;14(3):249. doi: 10.3390/jpm14030249 (PMC10971492; doi:10.3390/jpm14030249)

S1: Outcome of serum immunoglobulin A during and after apheresis sessions

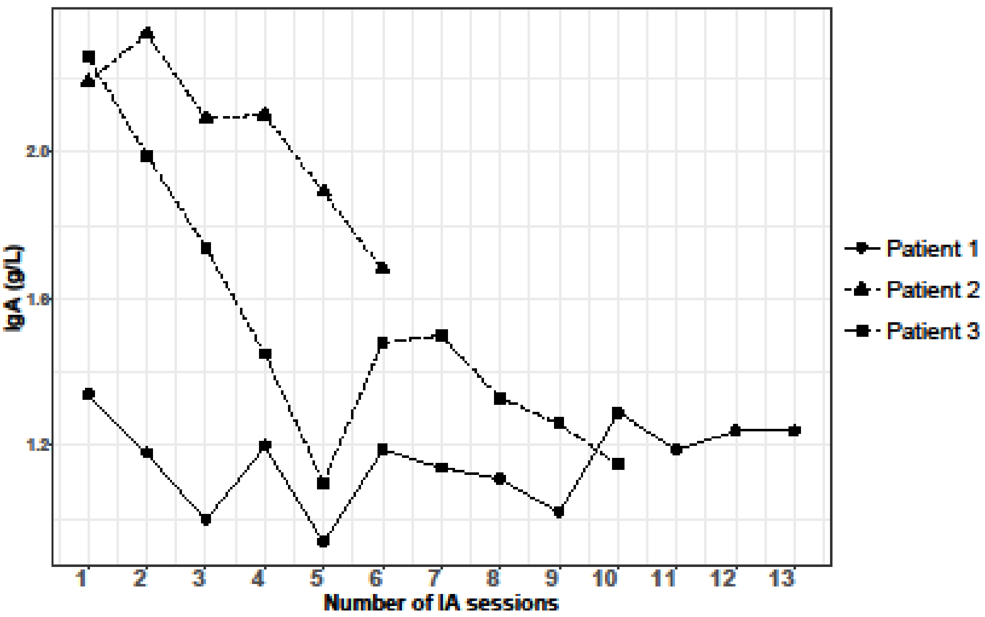

S2: Outcome of serum immunoglobulin M during and after apheresis sessions

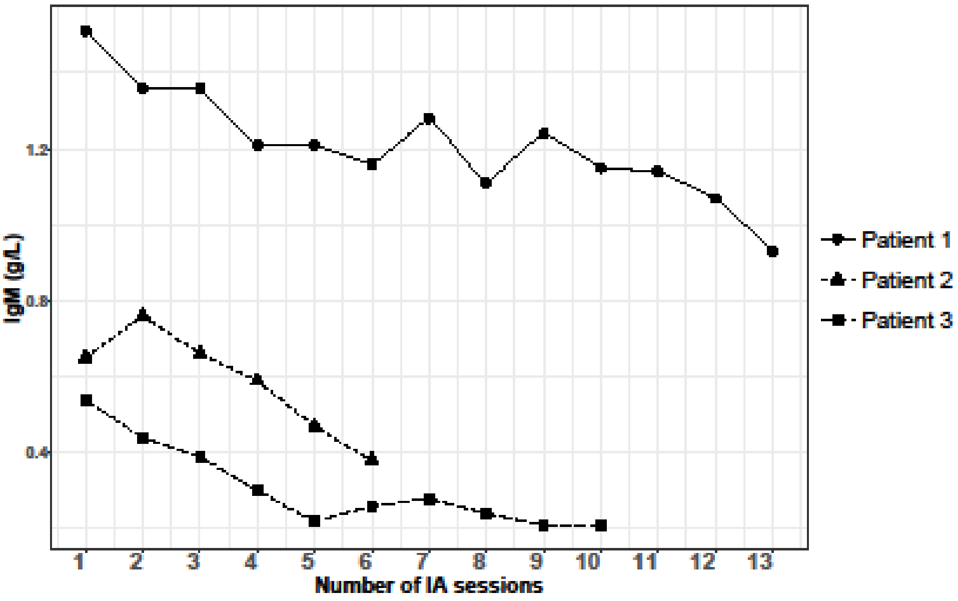

Supplement: Supplementary file 1 [file jpm-14-00249-s001.zip › jpm-2859671-supplementary.pdf]
